# Supplementary material for: Evaluation of Anopheline Diversity and Abundance across Outdoor Collection Schemes Utilizing CDC Light Traps in Nchelenge District, Zambia
Source: Insects. 2024 Aug 30;15(9):656. doi: 10.3390/insects15090656 (PMC11431859; doi:10.3390/insects15090656)
Supplement: Supplementary file 1 [file insects-15-00656-s001.zip › insects-3167483-supplementary.pdf]

Supplementary File for:

Title: Evaluation of Anopheline Diversity and Abundance across Outdoor Collection Schemes Utilizing CDC Light Traps in Nchelenge District, Zambia

Authors: Christine M. Jones, Ilinca I. Ciubotariu, Mary E. Gebhardt, James Sichivula Lupiya, David Mbewe, Mbanga Muleba, Jennifer C. Stevenson, and Douglas E. Norris

Tables

Table S1: Summary of models and their covariates for the N=20 tested negative binomial mixed models of female anopheline abundance per trap, which were ranked by cAIC with the model.sel function of the R package MuMIn [36]. Each '+' indicates that a covariate was used in the given model.

model rank	location	attractant	date	#ppl under net	#ppl in household	#ppl outside of net	#ITN	eave	roof	df	delta cAIC
1	+	+				+				7	0.0
2	+					+				5	0.5
3	+	+					+			7	5.6
4	+	+			+					7	7.1
5	+				+					5	7.2
6	+			+						5	7.6
7	+	+		+						7	7.7
8						+				4	12.8
9	+						+			5	26.2
10	+	+								6	28.7
11	+	+								6	28.7
12	+									4	29.0
13	+							+		6	30.4
14	+	+						+		8	30.5
15	+	+							+	9	33.8
16	+								+	7	34.1
17	+	+	+							14	34.3
18		+								5	45.5
19										3	46.1
20			+							11	50.7

Table S2: Summary of models and their covariates for the N=18 tested over-dispersed Poisson mixed models of the # species present in each trap, which were ranked by cAIC with the model.sel function of the R package MuMIn [36]. Each '+' indicates that a covariate was used in the given model.

model rank	location	date	attractant	#ppl under net	#ppl outside of net	#ITN	roof	eave	df	delta cAIC
1	+							+	5	0.0
2	+			+					5	1.0
3	+					+			5	1.4
4	+				+				5	3.0
5							+		5	3.0
6	+						+		5	3.0
7	+		+					+	7	3.9
8								+	4	5.2
9					+				4	6.7
10						+			4	9.1
11				+					4	9.4
12	+								4	14.7
13	+		+						6	18.8
14	+	+							12	23.2
15									3	25.1
16	+	+	+						14	28.6
17			+						5	29.1
18		+							11	33.3
